# Increased Expression of Galectin-3 in Skin Fibrosis: Evidence from In Vitro and In Vivo Studies

**DOI:** 10.3390/ijms232315319

**Published:** 2022-12-05

**Authors:** Teresa Peiró, Miriam Alonso-Carpio, Pilar Ribera, Patricia Almudéver, Inés Roger, Paula Montero, Severiano Marín, Javier Milara, Julio Cortijo

**Affiliations:** 1 Department of Nursing, Faculty of Nursing and Podiatry, University of Valencia, 46010 Valencia, Spain; 2 Plastic and Reconstructive Surgery Division, La Fe University and Polytechnic Hospital, 46026 Valencia, Spain; 3 Department of Pharmacology, Faculty of Medicine, University of Valencia, 46010 Valencia, Spain; 4 Department of Biotechnology, Polytechnic University of Valencia, 46022 Valencia, Spain; 5 CIBERES, Health Institute Carlos III, 28029 Madrid, Spain; 6 Faculty of Health Sciences, European University of Valencia, 46010 Valencia, Spain; 7 Plastic Surgery Unit, University General Hospital Consortium, 46014 Valencia, Spain; 8 Pharmacy Unit, University General Hospital Consortium, 46014 Valencia, Spain; 9 Research and Teaching Unit, University General Hospital Consortium, 46014 Valencia, Spain

**Keywords:** Galectin-3, skin, fibrosis

## Abstract

Skin fibrosis is a hallmark of a wide array of dermatological diseases which can greatly impact the patients’ quality of life. Galectin-3 (GAL-3) has emerged as a central regulator of tissue fibrosis, playing an important pro-fibrotic role in numerous organs. Various studies are highlighting its importance as a skin fibrotic diseases biomarker; however, there is a need for further studies that clarify its role. This paper aims to ascertain whether the expression of GAL-3 is increased in relevant in vitro and in vivo models of skin fibrosis. We studied the role of GAL-3 in vitro using normal human dermal fibroblasts (NHDF) and fibrocytes. In addition, we used a skin fibrosis murine model (BALB/c mice) and human biopsies of healthy or keloid tissue. GAL-3 expression was analyzed using real time PCR, Western blot and immunostaining techniques. We report a significantly increased expression of GAL-3 in NHDF and fibrocytes cell cultures following stimulation with transforming growth factor β1 (TGFβ1). In vivo, GAL-3 expression was increased in a murine model of systemic sclerosis and in human keloid biopsies. In sum, this study underlines the involvement of GAL-3 in skin fibrosis using several models of the disease and highlights its role as a relevant target.

## 1. Introduction

Fibrosis is characterized by an excessive accumulation of extracellular matrix (ECM) with tissue overgrowth, hardening and/or scarring as hallmarks of the process [1], which lead to organ injury, dysfunction and, ultimately, failure. In the skin, regulated ECM synthesis contributes to critical development processes such as scar formation and wound repair. However, an unregulated process can lead to skin fibrosis, where ECM accumulates in the dermis and leads to compromised function and altered structure [2]. Skin fibrosis is related to a wide array of dermatological diseases such as systemic sclerosis (SSc, also known as scleroderma), keloid formation, hypertrophic scars, graft-versus-host disease, among others [3,4], which can significantly impact the patients’ quality of life.

Cellular injury and chronic inflammation are considered key underlying causes of fibrotic tissue remodeling, which is characterized by subsequent activation of inflammatory cells, oxidative stress, uncontrolled fibroblast proliferation and ECM deposition [1,4]. Activated fibroblasts and myofibroblasts are the central effector cells of the fibrotic process, being the main producers of ECM. Myofibroblasts, the main producers of collagen, can originate from resident fibroblasts, epithelial and endothelial cells (epithelial/endothelial-mesenchymal transition; EMT/EndMT), bone marrow derived fibrocytes, pericytes or adipocytes [1,2]. Several pro-fibrotic growth factors have been described to play a role in inducing collagen synthesis, such as transforming growth factor β (TGFβ) [5] and connective tissue growth factor (CTGF) [6]. Particularly, TFGβ is considered a crucial regulator of fibrosis and is known to activate multiple oxidative stress-related genes involved in profibrotic pathways and to regulate cellular proliferation, differentiation, and ECM production [4,7]. Tissue fibrosis occurs in multiple organs which share common molecular pathways and key mediators. Thus, mechanisms such as epithelial and endothelial injury and dysfunction, abnormal proliferation of myofibroblasts and smooth muscle cells, and ECM deposition are described in fibrotic processes affecting different organs [8,9].

Galectin-3 (GAL-3) is extensively found throughout the body. Particularly in the skin, GAL-3 is expressed on keratinocytes in the basal and suprabasal layers, sweat glands, sebaceous glands associated with hair follicles, in hair follicles, in the extracellular matrix of the dermis, in proliferating fibroblasts, Langerhans cells, mast cells, and melanocytes [10]. Moreover, GAL-3 is also expressed in cells that migrate into the skin, such as dendritic cells, fibroblasts and monocytes [10]. Thus, GAL-3 appears as a key mediator in multiple skin diseases. GAL-3 plays a critical role in the development of the allergic inflammatory response in atopic dermatitis [11]. Recently, a study reported a significant decrease in skin inflammation in a porcine model through GAL-3 depletion using plasma perfusion by apheresis [12]. GAL-3 has also been involved in wound re-epithelialization in skin and in other tissues [13,14,15] and has been suggested as a possible adjuvant therapy for wound healing [16]. In addition, some authors have shown that GAL-3 is useful to protect against advanced glycation end products (AGEs) accumulation in wound healing [17]. GAL-3 deficiency has been suggested as a diagnostic biomarker of psoriasis, and the administration of recombinant GAL-3 has been described as a promising approach for its treatment [18]. In contrast, a GAL-3 inhibitor has shown a reduction in psoriasis when used in two Phase 2 clinical trials [19,20], evidencing a need to further clarify its role in this skin disease.

Considering the crucial role that GAL-3 plays in several skin diseases, it is not surprising that in the last years various studies have pointed out a role for this protein in some dermatological disorders that are associated with skin fibrosis. Relevantly, serum GAL-3 has been related with fibrosis and inflammation in SSc, particularly with skin fibrosis and proliferative vasculopathy [21,22]. Moreover, GAL-3 has been described as a reliable SSc biomarker, as its serum levels are related with advanced organ fibrosis, inflammation and clinical manifestations [23,24]. In addition, GAL-3 genetic variants could help to identify susceptibility to SSc and clinical features [25]. Recently, GAL-3 immunohistochemical expression in lesional skin of SSc patients has been associated with disease severity [26]. 

In addition, histological expression of GAL-3 has been recently described in keloids [27], a fibroproliferative skin disorder characterized by an excessive accumulation of ECM [3]. However, to the best of our knowledge, few in vitro or in vivo studies have been conducted to assess GAL-3 expression at a molecular level in relevant fibrotic models. Based on these observations, the aim of this study was to ascertain whether the expression of GAL-3 is increased both in in vitro and in vivo models that are significant for skin fibrosis. Thus, in this paper we analyzed the previously unexplored role of GAL-3 in skin fibrosis using normal human dermal fibroblasts (NHDF), fibrocytes, a skin fibrosis animal model and healthy skin tissue or keloid biopsies.

## 2. Results

### 2.1. GAL-3 Expression in Skin Fibrosis In Vitro Models with TGFB1 Stimulation

#### 2.1.1. Fibroblast to Myofibroblast Transition Model

**Figure 1:**
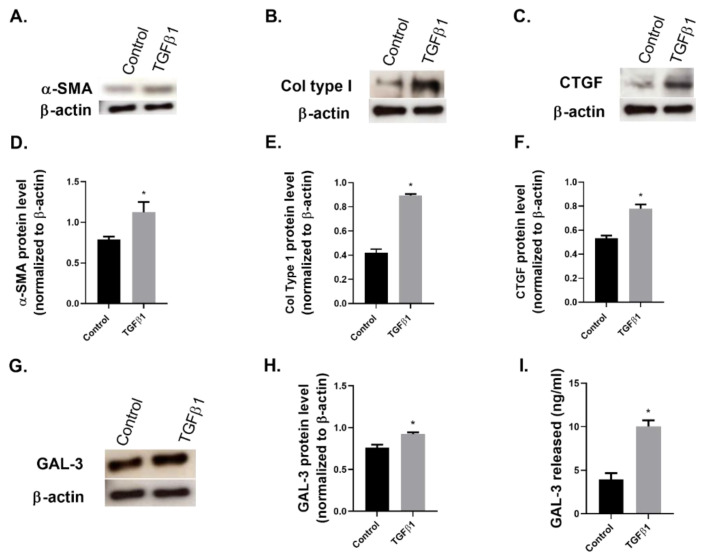
TGFβ1 induced expression of Galectin-3 in fibroblast to myofibroblast transition. Normal human dermal fibroblasts (NHDF) were stimulated with transforming growth factor β1 (TGFβ1) 10 ng/mL for 48 h. (**A**) Alpha smooth muscle actin (α-SMA), (**B**) Collagen type I (Col type I) and (**C**) connective tissue growth factor (CTGF) or β-actin loading control protein levels were assessed by Western blot. Densitometry analysis of (**D**) α-SMA, (**E**) Col type I and (**F**) CTGF expression from Western blot. (**G**) Galectin-3 (GAL-3) or β-actin loading control protein levels were assessed by Western blot. (**H**) Densitometry analysis of GAL-3 expression from Western blot. (**I**) GAL-3 release into supernatant was assessed by ELISA. The graphs present representative data from 3 experiments with *n* = 3 per group. Results are presented as mean ± SEM. Statistical analysis was performed with Mann–Whitney test (one-tailed) * *p* ≤ 0.05 vs. control.

In NHDF cell culture, TGFβ1 significantly increased the protein expression of the fibroblast to myofibroblast transition molecular marker alpha smooth muscle actin (α-SMA), the extracellular membrane (ECM) component, collagen type I (Col type I) and the pro-fibrotic factor connective tissue growth factor (CTGF) (Figure 1A–F). Importantly, in this fibrotic setting, TGFβ1 also increased the protein expression of GAL-3 and its secretion into the culture supernatant as measured by ELISA (Figure 1G–I).

#### 2.1.2. Human Fibrocytes Model 

**Figure 2:**
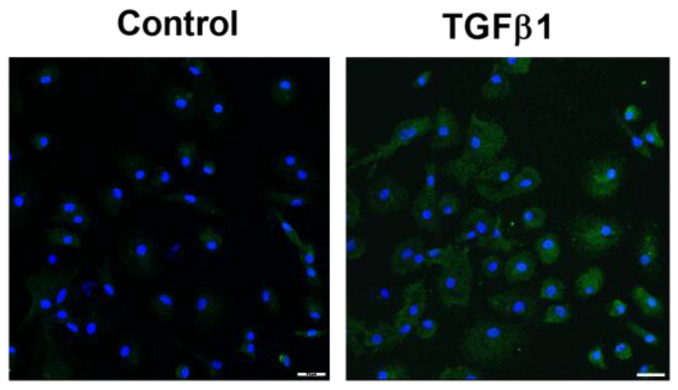
TGFβ1 induced expression of Galectin-3 in human fibrocytes. Fibrocytes were stimulated with transforming growth factor β1 (TGFβ1) 10 ng/mL for 48 h. Representative immunofluorescence microscope images of control and TGFβ1 10 ng/mL stimulated fibrocytes for GAL-3 immunostaining. DAPI, blue nuclei. Scale 25 µm.

Fibrocytes represent a cellular source of myofibroblasts in the fibrotic tissue [1,2]. In a cell culture of human fibrocytes, TGFβ1 induced the protein expression of GAL-3. This increase in GAL-3 protein was evident in the cytoplasm of TGFβ1-stimulated human fibrocytes, as evidenced by immunofluorescence (Figure 2).

### 2.2. GAL-3 Expression in In Vivo Models

#### GAL-3 Expression Is Increased in a Skin Fibrosis Murine Model

**Figure 3:**
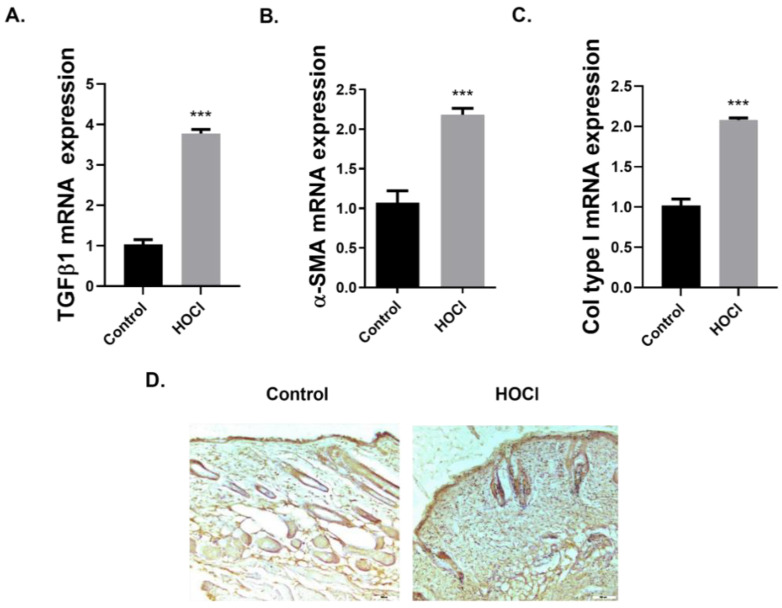
Skin fibrosis animal model. BALB/c mice were administered intra-dermal injections of 100 μL hypochlorite (HOCl) daily for 6 weeks. Mice were culled and levels of (**A**) Transforming growth factor β1 (TGFβ1), (**B**) alpha smooth muscle actin (α-SMA) and (**C**) collagen type I (Col type I) messenger RNA in fibrotic skin tissue were assessed by real-time PCR. Fibrotic skin tissue was embedded in paraffin and (**D**) α-SMA immunohistochemistry was performed. The graphs present data from one experiment with *n* = 7 (Control) and *n* = 8 (HOCl) mice per group. Results are presented as mean ± SEM. Statistical analysis was performed with two-tailed Student’s unpaired t-test for independent samples. *** *p* < 0.001 vs. control. Original magnification: ×10; Scale 200 µm.

We studied the expression of GAL-3 in a well-characterized murine model of skin fibrosis [28]. Following intradermal injections of hypochlorite (HOCl) for 6 weeks, we assessed the levels of fibrotic markers in the skin injury tissue. The gene expression of the fibrotic markers TGFβ1, α-SMA and Col type I was significantly increased in the HOCl-treated group (Figure 3A–C). Particularly, α-SMA expression was increased in the dermis of the HOCl-treated mice compared to the control group (Figure 3D). HOCl-treated mice also showed a thickening of both the epidermis and the dermis, a feature of skin fibrosis (Figure 3D). 

**Figure 4:**
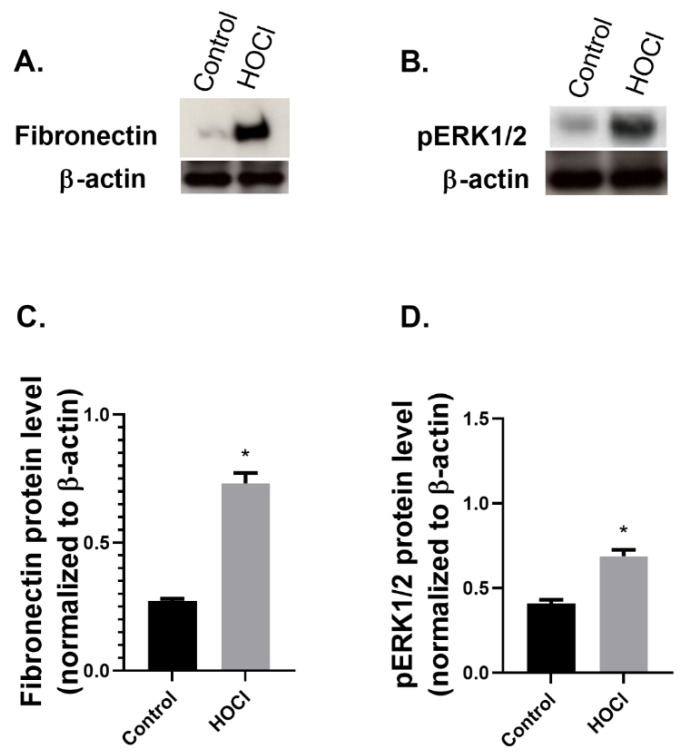
Increased molecular markers of fibrosis in skin fibrosis animal model. BALB/c mice were administered intra-dermal injections of 100 μL hypochlorite (HOCL) daily for 6 weeks. Mice were culled and (**A**) Fibronectin and (**B**) pERK1/2 or β-actin loading control protein levels were assessed by Western blot in fibrotic skin tissue. Densitometry analysis of (**C**) Fibronectin and (**D**) pERK1/2 expression from Western blot. The graphs present representative data from one experiment with *n* = 7 (Control) and *n* = 8 (HOCL) mice per group. Results are presented as mean ± SEM. Statistical analysis was performed by Mann–Whitney test (one-tailed). * *p* ≤ 0.05 vs. control.

We then assessed the protein levels of the fibrotic marker fibronectin, which was significantly increased compared to the control group (Figure 4A,C). Furthermore, the protein expression of the TGFβ-related protein pERK1/2 was also increased following HOCl administration (Figure 4B,D). 

**Figure 5:**
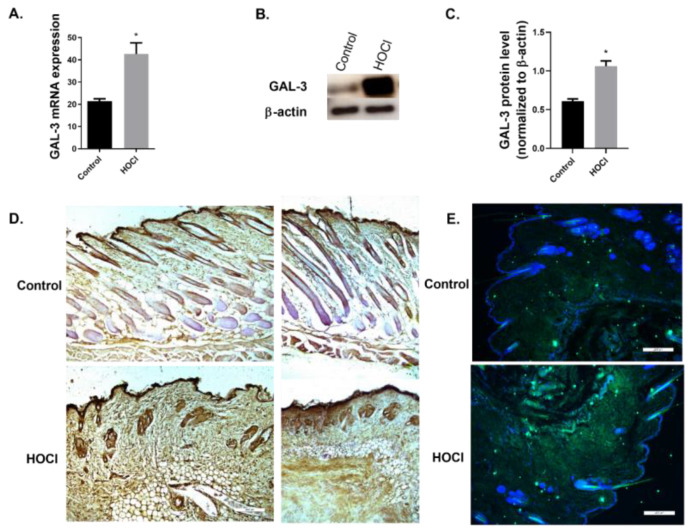
Galectin-3 expression is increased in a skin fibrosis animal model. BALB/c mice were administered intra-dermal injections of 100 μL hypochlorite (HOCl) daily for 6 weeks. Mice were culled and levels of (**A**) Galectin-3 (GAL-3) messenger RNA in fibrotic skin tissue were assessed by real-time PCR. (**B**) Western blot depicting GAL-3 or β-actin loading control. (**C**) Densitometry analysis of GAL-3 expression from Western blot. (**D**) Representative immunohistochemistry microscope images of skin sections from control and HOCl for GAL-3 immunostaining. Original magnification: ×10; Scale: 200 µm. (**E**) Representative immunofluorescence microscope images of skin sections from control and HOCl for GAL-3 immunostaining. DAPI, blue nuclei. Scale 200 µm. The graphs present representative data from (**A**) one experiment with *n* = 7 (Control) and *n* = 8 (HOCl) mice per group, (**B**,**C**) one experiment with *n* = 7 (Control) and *n* = 8 (HOCl) mice per group. Results are presented as mean ± SEM. Statistical analysis was performed by Mann–Whitney test (one-tailed). * *p* ≤ 0.05 vs. control.

In this murine model of skin fibrosis, both gene and protein expression of GAL-3 are significantly increased compared to the control group (Figure 5A–C). We then assessed the location of GAL-3 protein in the skin tissue using immunohistochemistry and immunofluorescence. GAL-3 is expressed in the epidermis of both control and HOCl-treated mice. GAL-3 expression is increased in the dermis of HOCl-treated mice compared to the control group (Figure 5D,E).

### 2.3. GAL-3 Expression in Skin Biopsies of Human Keloids

#### 2.3.1. Expression of Fibrotic Markers Is Increased in Human Keloids Compared to Human Healthy Skin Tissue

**Figure 6:**
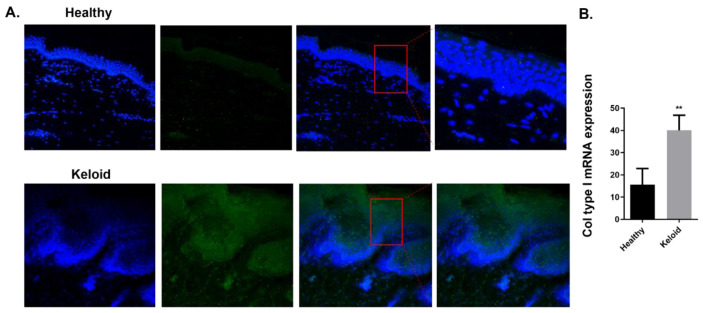
Increased molecular markers of fibrosis in skin biopsies of human keloids. Human skin tissue was obtained from healthy controls and patients with keloids. (**A**) Representative immunofluorescence microscope images of skin sections from healthy and keloid samples for alpha smooth muscle actin (α-SMA) immunostaining. DAPI, blue nuclei. Original magnification: ×20; Scale 100 µm. Levels of (**B**) Collagen type I (Col type I) messenger RNA were assessed by real-time PCR. (**B**) The graphs present data from *n* = 8 (Healthy) and *n* = 8 (Keloid) samples. Results are presented as mean ± SEM. Statistical analysis was performed by Mann–Whitney test (one-tailed). ** *p* < 0.01 vs. healthy control.

We then analyzed the expression of fibrotic markers in human keloids compared to human healthy skin tissue. Immunofluorescence of the mesenchymal marker α-SMA evidences its increased expression in keloid epidermis and dermis compared to healthy tissue (Figure 6A). Furthermore, the gene expression of the ECM component Col type I was increased in keloids compared to the healthy tissue (Figure 6B).

#### 2.3.2. Expression of GAL-3 Is Increased in Human Keloids Compared to Human Healthy Skin Tissue

**Figure 7:**
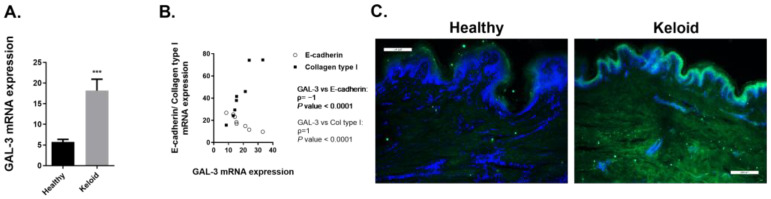
Galectin-3 expression is increased in skin biopsies of human keloids. Human skin tissue was obtained from healthy controls and patients with keloids. Levels of (**A**) Galectin-3 (GAL-3) messenger RNA were assessed by real-time PCR. (**B**) Correlation between levels of GAL-3 messenger RNA and E-cadherin messenger RNA or Collagen type I messenger RNA. (**C**) Representative immunofluorescence microscope images of skin sections from healthy and keloid samples for GAL-3 immunostaining. DAPI, blue nuclei. Scale 200 µm. (**A**,**B**) The graphs present data from *n* = 8 (Healthy) and *n* = 8 (Keloid) samples. Results are presented as mean ± SEM. Statistical analysis was performed by Mann–Whitney test (one-tailed) and with Spearman correlation. *** *p* < 0.001 vs. healthy control; ρ indicates Spearman’s rank correlation coefficient.

We then analyzed the expression of GAL-3 in skin biopsies. GAL-3 gene expression is increased in keloid tissue compared to the healthy control group (Figure 7A) and shows a strong positive correlation with Col type I gene expression and a strong negative correlation with E-cadherin gene expression (Figure 7B). GAL-3 immunofluorescence evidences its increased expression in both epidermis and dermis of keloid samples (Figure 7C).

## 3. Discussion

In this paper we analyzed the previously unexplored role of GAL-3 in several in vitro and in vivo models of skin fibrosis. We report an increased expression of GAL-3 in currently employed models, pointing out its role as an important regulator of the skin fibrotic process. 

As mentioned earlier, the pro-fibrotic factor TGFβ1 is a well-recognized regulator of fibrosis in multiple organs [4,7], and this is partially mediated through its role in supporting myofibroblast activation [29,30]. Fibroblasts and myofibroblasts are key cells in the initiation and perpetuation of tissue fibrogenesis: fibroblast to myofibroblast transition has been described as a hallmark of fibrosis and TGFβ1 is known to trigger this process [31,32]. Specifically, the pathogenesis of various skin fibrotic diseases, such as SSc, involves fibroblast dysfunction and an excessive number of myofibroblasts and TGFβ1 have been identified as a regulator of pathological fibrogenesis in this disease, in which collagen accumulation is another major feature [31]. Additionally, in keloids, TGFβ also stimulates fibroblast proliferation and collagen synthesis [3]. In our study, the stimulation of NHDF with TGFβ1 increased the protein expression of the myofibroblast marker α-SMA, as well as Col type I and CTGF. Interestingly, in this fibrotic setting the expression of GAL-3 protein was also significantly increased. The properties of GAL-3 as a strong fibroblast mitogen in vitro, stimulating DNA synthesis and proliferation of fibroblasts in multiple cultures and tissues, have been described in the literature [33]. More recently, it has been reported that GAL-3 reversed TGFβ1-mediated ECM deposition in pulmonary artery fibroblasts [34]. In addition, blocking GAL-3 was described to inhibit myofibroblast activation and decrease collagen type I expression in vitro and in vivo, and diminish liver fibrosis [35]. Furthermore, GAL-3 has been described as a critical mediator of TGFβ-induced fibrosis in liver and also in lungs, using GAL-3 knockout (KO) mice, which show fewer fibrosis and a diminished synthesis and mediation of TGFβ1 [35,36,37]. In the same line, lung fibroblasts from GAL-3 KO mice show impaired myofibroblast differentiation and reduced collagen type I synthesis in vitro in response to TGFβ1 [36]. However, a study in skin that describes impaired re-epithelialization in GAL-3 KO mice following dermal wounding found an unexpected GAL-3 downregulation by TGFβ1 in vitro in WT dermal fibroblasts, and in human chronic wound edge dermal fibroblasts compared to noninvolved fibroblasts of the same patients [38]. These authors suggested a tissue and cell-specific role for GAL-3 in fibrosis. A recent paper measured fibroblast GAL-1 and GAL-3 expression in affected skin of patients with SSc, and reported a lower expression in scleroderma lesional skin compared with a normal control [26]. These results contrast with our results in NHDF subjected to in vitro fibrotic conditions. This discrepancy could be explained due to the pathological or normal condition in these cells and would need further investigation. 

Fibrocytes, circulating bone marrow derived cells that produce matrix proteins such as collagen, have been related to fibrotic lesions in the skin, lungs and tumors [39]. Fibrocytes are a cellular source for myofibroblasts [1,2], and have been described to play an important role in the development of fibrotic skin lesions such as SSc, hypertrophic scars and keloids [39]. Subsequently, we stimulated cultured human fibrocytes with TGFβ1, which has been described to produce an increase in the expression of the myofibroblast marker α-SMA in these cells [40,41]. We found that TGFβ1 also induced the protein expression of GAL-3 in the cytoplasm of cultured human fibrocytes. To our knowledge, this is the first study that addresses GAL-3 expression in TGFβ1-stimulated human fibrocytes. 

In addition to cell cultures, we studied the expression of GAL-3 in a murine model of skin fibrosis which represents a model of human SSc, as it recapitulates inflammation, skin and pulmonary fibrosis in this disease evidencing a direct role for reactive oxygen species (ROS) in these processes [28]. Oxidative stress pathways have been described in SSc, and also in other skin fibrotic disorders such as hypertrophic scars and keloids, among others [4]. In our study, intradermal injections of HOCl during the 6 weeks increased the gene expression of the fibrotic markers TGFβ1, α-SMA and Col type I. Our results corroborate the increased expression of the myofibroblast marker α-SMA and the ECM component Col type I, as previously described in this model [28,42,43,44]. Several previous works did not find significant differences in the expression of TGFβ1 in mice exposed to HOCl versus control mice [28,42]; however, we found a significant increase in TGFβ1 gene expression in HOCl-treated mice compared to the control group. Similarly, in recent studies that addressed the timing and molecular characterization of the fibrotic process in this model, the authors described a strong expression of TGFβ1 in HOCl-treated mice at days 21 and 42 post-injection [43,44]. Evidence from several in vitro and in vivo studies suggests that TGFβ plays a key role in the development of tissue fibrosis in SSc [31,45], but the specific literature regarding the presence and role of TGFβ1 in this SSc murine model is contradictory. 

In our experiments, the protein levels of the ECM glycoprotein fibronectin were also increased compared to the control group. Furthermore, the protein expression of the TGFβ-related molecular marker pERK1/2 was also increased following HOCl administration. The marker pERK1/2 has been implicated in the pathways of TGFβ1-induced fibrosis in SSc [4,31,46].

It is worth noting that in this murine model of skin fibrosis, both gene and protein expression of GAL-3 are significantly increased compared to the control group. Furthermore, GAL-3 protein expression is increased in the dermis of HOCl-treated mice, as assessed by immunohistochemistry and immunofluorescence techniques. To the best of our knowledge this is the first paper that describes GAL-3 expression in the SSc murine model and highlights its use to further assess the molecular mechanisms in which GAL-3 is involved in skin fibrosis. Various studies have reported increased GAL-3 serum levels in SSc patients compared to healthy subjects [21,47], although in contrast, another study reported that serum GAL-3 levels were significantly lower in patients with diffuse cutaneous SSc than in healthy subjects and limited cutaneous SSc [22]. A recent study has described a lower expression of GAL-3 in scleroderma lesional skin compared to normal controls, although the authors report a significant correlation between higher GAL-3 expression in fibroblasts from lesional skin of SSc patients and severe disease [26]. Thus, studies are pointing out the involvement of GAL-3 in SSc skin fibrosis, but there is a need for further research with models of the disease to clarify its role and the related molecular mechanisms.

Concerning fibrotic human skin disease, we also studied keloid biopsies. We found an increased expression of the fibrotic marker α-SMA in these biopsies compared to healthy skin tissue, in line with the presence of α-SMA-positive myofibroblasts in keloids previously reported [48,49]. We also found an increased gene expression of Col type I, the main ECM component in keloid tissue [3]. 

In this fibrotic context that characterizes keloid pathology, we found an increased GAL-3 gene expression in keloid epidermis and dermis compared to healthy tissue. Moreover, we describe a strong positive correlation between GAL-3 and Col type gene expression, and a strong negative correlation between GAL-3 and E-cadherin gene expression. Epithelial–mesenchymal cross-talk has also been described between keratinocytes and dermal fibroblasts [50], and a distinctive feature of this process is the alteration in cell surface markers from an epithelial phenotype to an upregulation of mesenchymal markers. Thus, a decreased expression of E-cadherin is considered a hallmark of EMT [51]. Previous studies have described decreased E-cadherin levels in keloid tissue [52,53], in line with our results.

Our results corroborate a recent study that has described the presence of GAL-3 in keloid biopsies exclusively by immunostaining [27]. The authors speculate that GAL-3, together with GAL-1 and other ECM molecules produced by fibroblasts and by immune cells, counteract the inflammatory response in keloids. However, further mechanistic studies that address the GAL-3-related pathways are required to be able to elucidate its role in the disease. Skin fibrosis deserves more research in order to elucidate possible mechanisms that promote it. Among these mechanisms, the role of mast cells and their inflammatory mediators has been included in some studies [54] and the results about their importance for skin fibrosis are currently in debate [55,56]. Mast cells also express GAL-3 and its specific role in skin fibrosis has not been assessed. The ECM, fibroblasts, and inflammatory mediators have been traditionally investigated separately, but it has become increasingly necessary to consider them as parts of a complex and tightly regulated system that becomes dysregulated in fibrosis [57].

This study contributes to this area describing GAL-3 expression in different cell types and relevant in vitro and in vivo models for skin fibrosis, in order to highlight the importance of this molecule and set the basis for future studies which assess the mechanisms related to GAL-3 in skin fibrosis. To conclude, our results point out the involvement of GAL-3 using several models of skin fibrosis and highlight this molecule as an important mediator of skin fibrotic diseases. Furthermore, these models represent useful tools to study this target in depth, as the mechanisms involving GAL-3 in skin fibrosis are still not completely understood and the need for anti-fibrotic treatments remains. 

## 4. Materials and Methods

### 4.1. Cell Cultures

Normal human dermal fibroblasts (NHDF) (Promocell, Heidelberg, Germany) were cultured with Dulbecco’s Modified Eagle’s Medium (DMEM) High Glucose medium (Biowest, Riverside, CA, USA) supplemented with 10% Fetal Bovine Serum (FBS) (Hyclone, GE Healthcare, Uppsala, Sweden), penicillin-streptomycin mixture (Lonza; Basel, Switzerland) and amphotericin B (Hyclone, Uppsala, Sweden).

Fibrocytes were obtained from buffy coat samples of healthy donors in the University General Hospital Consortium of Valencia (CHGUV). The protocol was approved by the local research and independent ethics committee and informed written consent was obtained from each participant. To isolate fibrocytes, phosphate buffered saline (PBS) (Sigma-Aldrich, Madrid, Spain) was added to the buffy coat sample (1:1), and the mixture was poured on a falcon tube with Ficoll (GE Healthcare, Uppsala, Sweden). The mixture was then centrifuged (1300 rpm, 35 min) and the white layer was collected and mixed with physiological saline solution NaCl 0.9% (B. Braun; Melsungen, Germany) and PBS. The mixture was centrifuged (2000 rpm, 10 min) and the pellet was resuspended in DMEM supplemented with 20% FBS, 1% penicillin-streptomycin mixture and 1% amphotericin B.

### 4.2. Skin Biopsies: Healthy vs. Keloid Human Tissue

Human skin tissue was obtained from the Plastic Surgery Unit of the CHGUV. Human keloid biopsies were obtained from 2 women and 6 men, ranging in age from 35 to 56 years, with an average age of 49.5 years. Human healthy skin tissue (control samples) was obtained from patients undergoing aesthetic surgery procedures/without any skin disease (*n* = 8). The protocol was approved by the local research and independent ethics committee of the CHGUV (CHGUV/16/1/2016) and performed according to the Declaration of Helsinki. Informed written consent was obtained from each participant.

### 4.3. Skin Fibrosis Animal Model

This study was carried out in accordance with the guidelines of the Committee of Animal Ethics and Well-being of the University of Valencia (Valencia, Spain; CEA 2017/VSC/PEA/00062) following ARRIVE guidelines. Male BALB/c mice, 12 weeks old, were purchased from Harlan UK Ltd. (Derby, United Kingdom). All mice were kept in specific pathogen-free conditions, were provided autoclaved food, water and bedding and were housed under controlled light and temperature conditions (relative humidity 55 ± 10%; temperature 22 ± 3 °C; 15 air cycles/ per hour; 12/12 h Light/Dark cycle).

Cutaneous fibrosis induced by intra-dermal injections of hypochlorite (HOCl) represents a general model of skin fibrosis associated with oxidative stress, a critical mediator in promoting skin fibrosis pathogenesis, and may be best used as a model tool to study SSc [28,42,58]. BALB/c mice were anaesthetized with isoflurane (Aerrane^®^) and administered daily an intradermal injection of HOCl at 0.1 mg/mL (dissolved in 100µL of physiological saline solution) in the back of the mouse during the 6 weeks. Sham treated mice received the identical volume of physiological saline solution instead of HOCl. At the end of the protocol, mice were sacrificed by a lethal injection of sodium pentobarbital followed by exsanguination. Skin tissue was processed for experimental histological or molecular biology studies. For each experiment, the number of mice per group are specifically defined within the figure legends detailing the respective studies.

### 4.4. Real-Time RT-PCR

Real-time RT-PCR analysis was used to detect changes in mRNA expression in mice skin tissue from the in vivo model and in human healthy and keloid tissue. Total RNA was obtained using the TriPure^®^ Isolation Reagent (Roche, Indianapolis, IN, USA) and with the TissueLyser II (QIAGEN, Hilden, Germany). The tissue sample was disrupted through high-speed shaking (30 Hz, 3 min) in plastic tubes with TriPure^®^ Isolation Reagent and a bead. The sample was centrifuged (10,000 rpm, 10 min) and the supernatant was collected. The integrity of the extracted RNA was confirmed with Bioanalyzer 2100 (Agilent, Palo Alto, CA, USA). Reverse transcription was performed in 300 ng of total RNA using the TaqMan reverse transcription reagents kit (Applied Biosystems, Waltham, MA, USA). The resulting cDNA was amplified with specific primers and probes predesigned by Applied Biosystems for α-SMA (Mm00808218_g1), COL1 (Mm00801666_g1), TGFβ1 (Mm00441726_m1) and GAL-3 (Mm00802901_m1) for mice and α-SMA (Hs00559403_m1), COL1A1 (Hs00164004_m1), CTFG (Hs00170014_m1), E-cadherin (Hs01023894_m1) and GAL-3 (Hs00173587_m1) for human in a 7900HT Fast Real-Time PCR System (Applied Biosystems, Waltham, MA, USA) using TaqMan™ Gene Expression Master Mix (Applied Biosystems, Waltham, MA, USA). The expression of the target gene was shown as the fold increase or decrease relative to the expression of the endogenous control β-actin (Applied Biosystems, Waltham, MA, USA; Hs01060665 for human and Mm02619580_g1 for mouse). The mean value of the replicates for each sample was calculated and expressed as the cycle threshold (Ct). The level of gene expression was then calculated as the difference (ΔCt) between the Ct value of the target gene and the Ct value of β-actin. The fold changes in the target gene mRNA levels were designated 2-ΔCt.

### 4.5. Histological, Immunohistochemical and Immunofluorescence Studies

Murine and human skin tissues were fixed in formaldehyde 3.7–4.0% (PanReac, AppliChem, Darmstadt, Germany) for 48 h. The samples were dehydrated, paraffin wax embedded and cut into sections (5 μm thick).

The α-SMA and GAL-3 expressions were assessed by immunohistochemistry in paraffin-embedded sections of murine skin tissue. Sections were dewaxed by heat incubation (60 °C, 15 min), xylene (twice, 5 min each) and 100% ethanol (2 min). Sections were then incubated in methanol and hydrogen peroxide (174:6; 10 min) and incubated in PBS (3 times, 5 min each). Sections were then incubated in preheated 10 mM citrate, which was placed in boiling water for 10 min, washed with Milli-Q water (5 min) and blocked for 10 min at room temperature and avoiding light in Peroxidase Blocking Reagent from Master Polymer Plus Detection System (Master Diagnóstica, Granada, Spain). Following incubation, sections were washed in Tris-buffered saline (TBS; 0.05 M Tris, 0.15 M sodium chloride, pH 7.6, at 25 °C) (PanReac, AppliChem, Darmstadt, Germany) (3 times, 5 min each) and incubated overnight in a humid chamber (4 °C) with α-SMA (1/200) antibody (Sigma-Aldrich, Madrid, Spain) or anti-GAL-3 (1/200) antibody [A3A12] (Abcam, Cambridge, United Kingdom) in 0.1% bovine serum albumin (BSA) (Sigma-Aldrich, Madrid, Spain) in PBS. Following overnight incubation, sections were incubated for 30 min with the Master Polymer Plus HRP (Master Diagnóstica, Granada, Spain) at room temperature and avoiding light. Sections were washed in TBS (3 times, 5 min each) and incubated with DAB Chromogen Concentrate in DAB Substrate Buffer (Master Diagnóstica, Granada, Spain) for 5 min at room temperature and avoiding light. Sections were washed with water (3 times, 5 min each), counterstained with hematoxylin (1/20), washed with water (3 times, 5 min each) and dehydrated through 90% and 100% ethanol to water (twice, 5 min each), and incubated in xylene (twice, 10 min each). Sections were mounted with DPX, and images were acquired with a Leica Microscope DM6000B (Leica Microsystems, Wetzlar, Germany).

The α-SMA or GAL-3 expressions were assessed by immunofluorescence in fibrocytes, in murine skin tissue and in human healthy and queloid tissue. Fibrocytes were washed with Dulbecco’s Phosphate Buffered Saline (DPBS) (Biowest, Riverside, CA, USA ) (3 times) and fixed with 100% methanol at −20 °C for 5 min. Cells were permeabilized in Triton X-100 (0.1% in PBS) for 8 min and blocked in 1% BSA in PBS for 1 h at room temperature. Following incubation, sections were incubated overnight in a humid chamber (4 °C) with anti-GAL-3 (1/200) antibody [A3A12] in 1% BSA in PBS. Following overnight incubation, sections were washed with PBS (3 times, 5 min each) and incubated for 1 h with a goat anti-mouse IgG Alexa Fluor 488 (1/200) secondary antibody (Invitrogen, Carlsbad, CA, USA) in 1% BSA in PBS at room temperature and avoiding light. Sections were washed with PBS (3 times, 10 min each) and incubated with DAPI (1/1000) (Sigma-Aldrich, Madrid, Spain) in 1% BSA in PBS for 2 min. Sections were washed with PBS (3 times, 10 min each) and were mounted with DPX. Fluorescence images were acquired with a Leica Microscope DM6000B (Leica Microsystems, Wetzlar, Germany).

Tissue sections (5 µm) were dewaxed by heat incubation (60 °C, 15 min), xylene (twice, 10 min each) and 100% ethanol. Sections were permeabilized in Triton X-100 (0.1% in PBS) for 10 min, washed in PBS (3 times, 5 min each) and blocked for 10 min at room temperature and avoiding light in Peroxidase Blocking Reagent from Master Polymer Plus Detection System (Master Diagnóstica, Granada, Spain). Following incubation, sections were washed in TBS (3 times, 5 min each) and incubated overnight in a humid chamber (4 °C) with α-SMA (1/200) antibody or anti-GAL-3 (1/200) antibody in 0.1% BSA in PBS. Following overnight incubation, sections were washed with PBS (3 times, 5 min each) and incubated for 1 h with a goat anti-mouse IgG Alexa Fluor 488 (1/200) secondary antibody (Invitrogen, Carlsbad, USA) in 0.1% BSA in PBS at room temperature and avoiding light. Sections were washed with PBS (3 times, 10 min each) and incubated with DAPI (1/1000) (Sigma-Aldrich, Madrid, Spain) in 1% BSA in PBS for 2 min. Sections were washed with PBS (3 times, 10 min each) and were mounted with DPX. Fluorescence images were acquired with a Leica Microscope DM6000B (Leica Microsystems, Wetzlar, Germany).

### 4.6. Western Blotting Analysis

Western blotting analysis was used to detect changes in protein expression in mouse skin tissue from the in vivo model and in human NHDF.

Skin tissue was homogenized through high-speed shaking (30 Hz, 10 min) with the TissueLyser II (QIAGEN) using a lysis buffer (0.02 M HEPES, 0.001 M EDTA, 0.001 M EGTA, 0.4 M NaCl and 21.1% glycerol) with a complete inhibitor cocktail and phenylmethylsulfonyl fluoride (0.1 mM PMSF). The sample was centrifuged, and the supernatant was collected. Cells were scraped from a confluent petri dish with the same lysis buffer. Subsequently, both tissue and cell samples were lysed following a thermal lysis protocol followed by an incubation in ice with the detergent nonidet P-40 (1%). Samples were then centrifuged (10,000 rpm, 20 min, 4 °C) and the supernatant was collected. In order to quantify the level of protein in each sample to ensure equal protein loading, the Pierce™ BCA Protein Assay Kit (Thermo Fisher, Waltham, MA, USA; catalog no. 23227) was used following the manufacturer’s instructions.

Polyacrylamide gel electrophoresis was used to separate the proteins according to their molecular weight. Briefly, 12 µg of (denatured) proteins, along with a molecular weight protein marker (Spectra™ Multicolor Broad Range Protein Ladder) (ThermoFisher Scientific; Waltham, MA, USA; catalog no. 26623), were loaded onto 4–15% Mini-PROTEAN^®^ TGX™ Gel (Biorad, Hercules, CA, USA; catalog no. 4561086) or 4–20% Mini-PROTEAN^®^ TGX™ Gel (Biorad; catalog no.4561094), and run through the gel applying 90 V (30 min) and 300 V (30 min).

Following protein separation by electrophoresis, proteins were transferred from the gel to a polyvinylidene difluoride membrane using a semi wet-blotting method (Trans-Blot^®^ TurboTM Transfer System, BioRad). Membranes were blocked (5% BSA in PBS containing 0.1% Tween20 (PBS-T)) for 1 h at room temperature and were incubated overnight at 4 °C with the following primary antibodies diluted in 5% BSA in PBS-T: α-SMA (1/1000) antibody (42 KDa, monoclonal antibody) (Sigma-Aldrich, Madrid, Spain), collagen type I (1/1000) antibody (139 KDa, polyclonal antibody) (Novus Biologicals, Centennial, USA), connective tissue growth factor (CTGF) (1/200) antibody (38 KDa, polyclonal antibody) (Santa Cruz Biotechnology, Dallas, USA), phosphorylated ERK1/2 [Thr202, Tyr204] (1/1000) antibody (42–44 KDa, monoclonal antibody)(Invitrogen, Carlsbad, USA); fibronectin (1/1000) antibody (230–250 KDa, polyclonal antibody) (Invitrogen Carlsbad, USA), anti-Galectin 3 (GAL-3) [A3A12] (1/2000) antibody (30 KDa, monoclonal antibody) Abcam, Cambridge, United Kingdom) and normalized to anti β-actin (1/2000) antibody—loading control (40 KDa, monoclonal antibody) (Sigma-Aldrich, Madrid, Spain). Membranes were subsequently washed (PBS-T) and incubated for 1–2 h at room temperature with donkey anti-mouse IgG-horseradish peroxidase (HRP) secondary antibody (polyclonal antibody) (Invitrogen, Carlsbad, USA)) or donkey anti-rabbit IgG-HRP secondary antibody (polyclonal antibody) (Invitrogen, Carlsbad, USA) diluted in 5% BSA in PBS-T. Membranes were subsequently washed (PBS-T) and reactivity was visualized by using the ECL™ Prime Western Blotting System (GE Healthcare, Uppsala, Sweden) and Hyperfilm™ ECL™ films (GE Healthcare, Uppsala, Sweden), following manufacturer’s instructions. The densitometry analysis of the films was performed using the Image J 1.42q software (available at https://imagej.nih.gov/ij/, USA; accessed on 15 September 2022). Results of target protein expression are expressed as the ratio of the densitometry from the β-actin endogenous control.

### 4.7. ELISA

The levels of Gal-3 in the cell-free supernatant of cultured NHDF were measured using commercially available Galectin 3 Human ELISA kit (Invitrogen Carlsbad, USA) following the manufacturer’s instructions.

### 4.8. Statistical Analysis

Statistical analysis of the data was carried out by non-parametric (cell and human studies) and parametric (animal studies) analysis as convenient, where *p* ≤ 0.05 was considered statistically significant. Regarding non-parametric analysis, as the comparisons concerned two groups, between group differences were analyzed by the Mann–Whitney test. In addition, a Spearman correlation was computed between GAL-3 and Collagen type I expression (r = 1; *p* < 0.0001) and between GAL-3 and E-cadherin expression (r = −1; *p* < 0.0001). The statistical analyses of the results from animal experiments were carried out by parametric analysis, since normal distribution for each data set was confirmed by Shapiro–Wilk test and Kolmogorov–Smirnov test. Therefore, in these experiments two-group comparisons were analyzed using the two-tailed Student’s unpaired t-test for independent samples. Data are displayed as mean ± SEM.

## Data Availability

Not applicable.
